# Assessment of the supraspinatus muscle fiber architecture with diffusion tensor imaging in healthy volunteers

**DOI:** 10.1186/s13244-024-01800-x

**Published:** 2024-10-09

**Authors:** Adrian Alexander Marth, Stefan Sommer, Georg Wilhelm Kajdi, Sophia Samira Goller, Thorsten Feiweier, Reto Sutter, Daniel Nanz, Constantin von Deuster

**Affiliations:** 1 Swiss Center for Musculoskeletal Imaging, Balgrist Campus AG, Zurich, Switzerland; 2 Department of Radiology, Balgrist University Hospital, Zurich, Switzerland; 3 Advanced Clinical Imaging Technology, Siemens Healthineers International AG, Zurich, Switzerland; 4 Siemens Healthineers AG, Erlangen, Germany; 5 Medical Faculty, University of Zurich (UZH), Zurich, Switzerland

**Keywords:** Diffusion tensor imaging, Pennation angle, Myoarchitecture, Rotator cuff

## Abstract

**Objectives:**

This study presents a framework for the calculation of supraspinatus (SSP) muscle pennation angles (PAs) from diffusion tensor imaging (DTI).

**Materials and methods:**

Ten healthy individuals (five females and five males; age 32.0 ± 4.7 years) underwent three sessions of 3-T MRI, including a stimulated echo acquisition mode DTI sequence. The imaging plane of the DTI sequence was angled along the intramuscular part of the SSP tendon. A custom-built software was developed and implemented to compute DTI-based PAs of the anterior and posterior SSP in relation to the orientation of the tendon. Subsequently, three readers measured PAs from the post-processed images. Test-retest reliability, inter-reader agreement, and intra-reader agreement of PA measurements were evaluated with intraclass correlation coefficients (ICCs).

**Results:**

The mean PA in the anterior SSP was 15.6 ± 2.1° and 10.7 ± 0.9° in the posterior SSP. MRI-derived PAs showed good to excellent test-retest reliability (ICC: 0.856–0.945), inter-reader agreement (ICC: 0.863–0.955), and intra-reader agreement (ICC: 0.804–0.955).

**Conclusion:**

PAs derived from DTI demonstrated good to excellent test-retest reliability, inter-reader agreement, and intra-reader agreement. We successfully implemented a highly standardized technique for evaluating PAs of the SSP muscle.

**Critical relevance statement:**

This proposed low-complex method might facilitate the increased use of the PA as a biomarker for pathological conditions of the rotator cuff.

**Key Points:**

- A low-complex method for measuring PAs of the SSP might help identify pathology early.
- The mean PA was 15.6 ± 2.1° and 10.7 ± 0.9° in the anterior and posterior SSP, respectively.
- ICCs were ≥ 0.856 for test-retest reliability, ≥ 0.863 for inter-reader agreement, and ≥ 0.804 for intra-reader agreement.

**Graphical Abstract:**

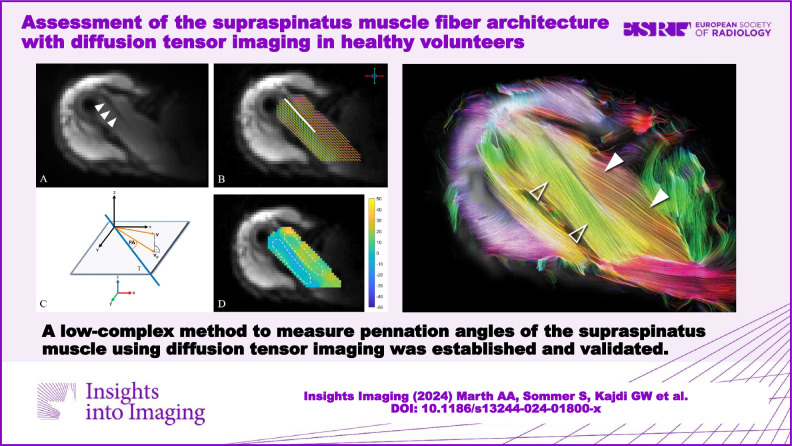

## Introduction

Pathologies of the rotator cuff are a significant health concern among the elderly population, and affect more than 25% of individuals over 60 years of age [1].

Quantitative assessment of the rotator cuff using various advanced imaging techniques has recently gained interest, with the aim of identifying biomarkers to assist in surgical management following rotator cuff tears [2, 3]. It has been observed that the pennation angle (PA) is sensitive to early pathological muscle changes (i.e., fatty infiltration and musculotendinous retraction) following rotator cuff injury [4, 5] and may be useful to predict the outcome following surgical cuff repair [6]. Ultrasound is the most commonly used modality to assess the PA of the skeletal muscle and is considered as clinical reference in vivo [7, 8]. However, current literature reports inconsistent PA values for the supraspinatus (SSP) muscle likely due to operator-dependency and difficulties in assessing the posterior muscle portion due to anatomical constraints [9–11].

Among the imaging techniques that are capable of visualizing muscular architecture, diffusion tensor imaging (DTI) has been successfully used to characterize the microstructure of healthy and pathological skeletal muscle [12, 13]. Due to the structural anisotropy of muscle fibers, it is possible to compute a diffusion tensor that reveals the primary direction of diffusion (the first Eigenvector of the tensor), and to provide information about diffusion anisotropy and diffusion magnitude. These data then facilitate the estimation of architectural parameters such as the PA [14–16] or by integrating the information from adjacent voxels, the three-dimensional (3D) reconstruction of the fiber trajectory known as “tractography”.

However, DTI has only recently been applied to the rotator cuff [17, 18] and is still considered challenging due to the complex muscle fiber organization and image artifacts caused by insufficient signal-to-noise ratio, which is highly influenced by respiratory motion, B1 inhomogeneities from shimming the neck and shoulder area, B0 artifacts near the lungs, and magnetic susceptibility variations within the region of interest. Furthermore, despite the fact that DTI has been available as a research tool for two decades, it still has not been implemented in routine clinical imaging.

Therefore, the objective of this study was to develop a low-complex two-dimensional (2D) method to measure PAs of the SSP muscle using DTI and to evaluate its test-retest reliability, inter-reader agreement, and intra-reader agreement.

## Material and methods

### Participants

This prospective study was approved by the local institutional review board (Cantonal Ethics Committee, Zurich, Switzerland). After providing written informed consent, ten healthy volunteers with no history of muscle injury, muscle disease, or previous shoulder surgery were enrolled, and demographic data was collected. Research was conducted in accordance with the ethical standards established by the institutional and/or national ethics committee and the 1964 Declaration of Helsinki and its subsequent amendments.

### MRI protocol

**Fig. 1:**
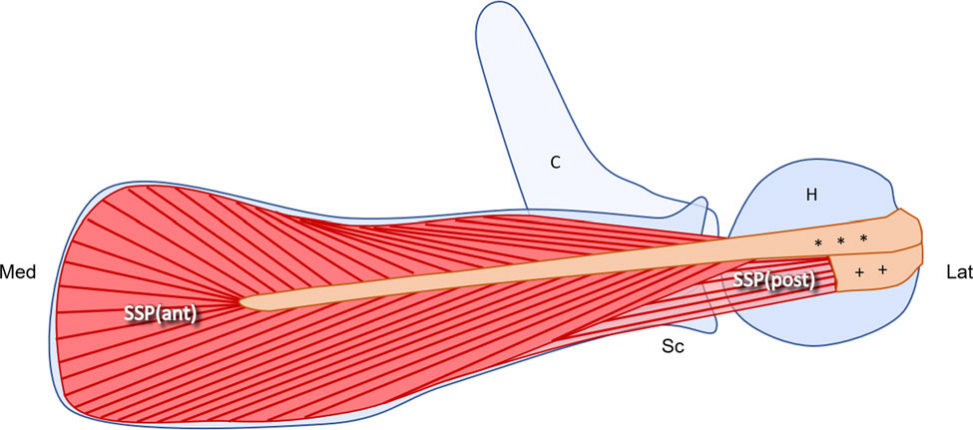
Schematic drawing depicting the anatomy of the SSP muscle of the right side at the level of the supraspinous fossa in an axial plane. The fusiform anterior portion of the supraspinatus (SSP_ant_) has a bipennate architecture with a long intramuscular tubular tendon that inserts on the anterior aspect of the superior facet of the greater tuberosity and lies mostly superficial to the posterior portion of the muscle (SSP_post_). The SSP_post_ has a “strap-like” appearance with fibers that are arranged in a more parallel pattern and inserts broadly on the posterior aspect of the superior facet of the greater tuberosity. C, clavicula; H, humeral head; Sc, scapula

MRI data were acquired were performed on a 3-T system (MAGNETOM Prisma, Siemens Healthineers AG, Erlangen, Germany) using a dedicated 16-channel shoulder coil. MRI scans were performed between May and August 2023, and comprised a total of three scans of the same shoulder for each volunteer, with an interval of seven days between the scan sessions, respectively. Patient positioning was performed for each examination according to the local clinical standard. For the planning of diffusion-weighted imaging, the maximum visible length of the intramuscular tendon of the anterior SSP muscle bundle was identified on the water-only images of a coronal oblique proton-density weighted turbo-spin-echo DIXON sequence. Figure 1 illustrates the anatomy of the SSP muscle, which consists of an anterior fusiform portion with a bipennate fiber arrangement that contains the dominant tendon and a posterior portion, which has a more parallel, “strap-like” arrangement [19]. The diffusion-weighted imaging parallel to the SSP tendon orientation was performed along 64 directions [20, 21] with three *b* = 0 s/mm^2^ images as reference using a monopolar stimulated echo acquisition mode research sequence (mixing time: 200 ms) with a single-shot echo-planar imaging readout. The sequence parameters are listed in Table 1.

**Table 1:** Sequence parameters of the imaging protocol

	Coronal oblique PD TSE DIXON	Axial oblique STEAM DTI
TR/TE, (ms)	2990/43	6700/36
Slice thickness, (mm)	4	3
Number of slices	21	15
Matrix size	640 × 640	256 × 256
FoV, (mm^2^)	160 × 160	192 × 192
Acceleration factor	2	2
Diffusion directions	–	64
Diffusion weightings	–	2 (0, 450)
Receive bandwidth, (Hz/Pixel)	1040	2005
Averages/*b*-value	–	1
Acquisition time, (min:s)	02:08	07:49

*DTI* diffusion tensor imaging, *FoV* field of view, *STEAM* stimulated echo acquisition mode

### MRI analysis

**Fig. 2:**
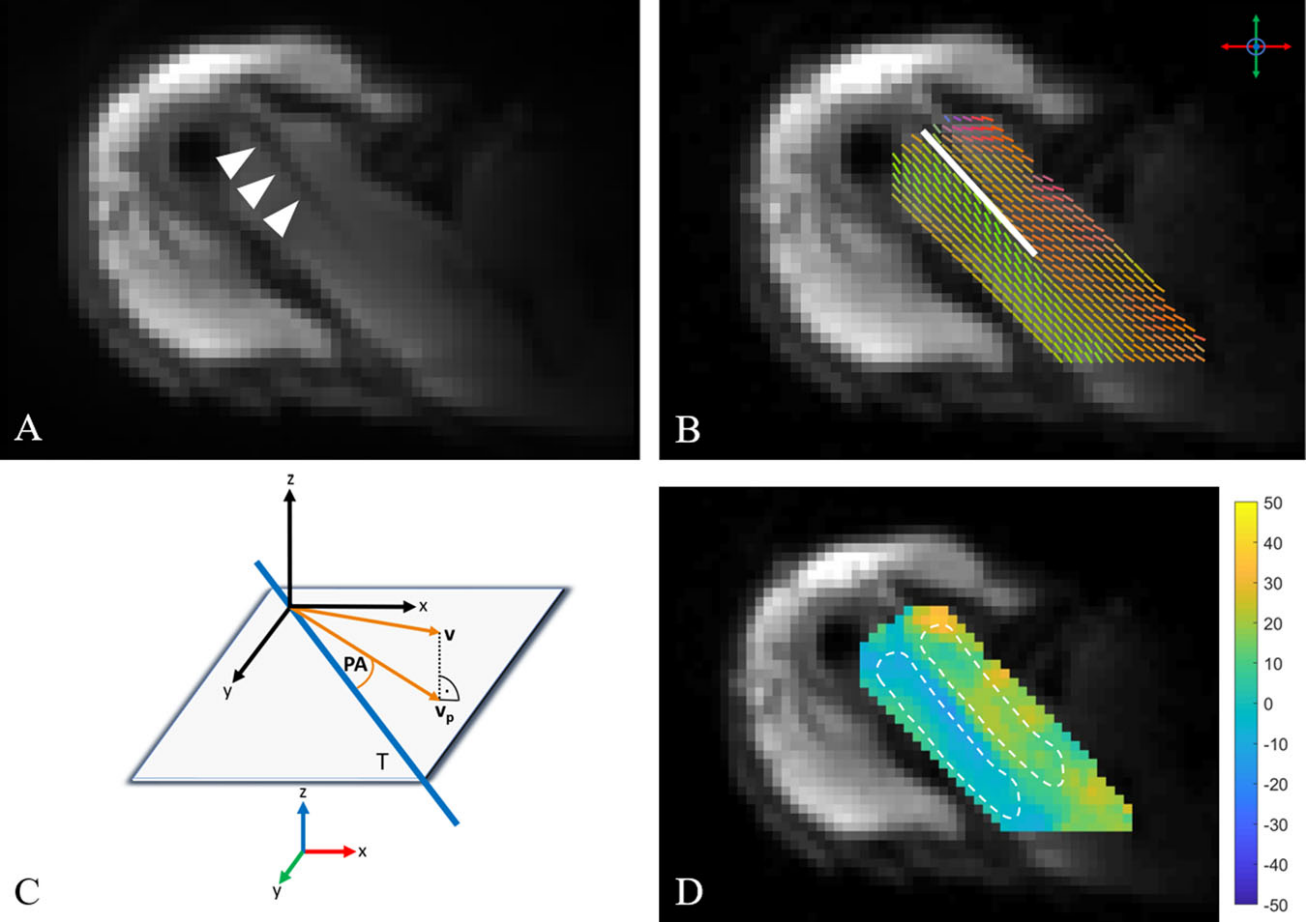
Following acquisition and registration, images were processed using an in-house developed Matlab script: first, the SSP tendon (arrowheads, **A**; white line, **B**) was manually drawn into the *b* = 0 s/mm^2^ images, followed by tensor fitting. Color-coded directions of the first Eigenvector in each voxel are shown, with green color indicating anteroposterior orientation, red color indicating mediolateral orientation, and blue color indicating superoinferior orientation (**B**). The mathematical operation to obtain the 2D PA in relation to the SSP tendon (T) is visualized in **C**. The first Eigenvector (*v*) is projected in the transverse plane (*v*_p_), followed by a computation of the angle relative to the SSP tendon. Freehand ROIs (dashed lines) were then drawn in the anterior SSP portion (ROI_ant_) and posterior SSP portion (ROI_post_) in the PA maps (**D**). Images of the right shoulder of a 31-year-old male subject in the oblique axial plane are shown

Geometric inconsistencies due to eddy-current-induced distortions or physiological motion were corrected by non-rigid image registration [22]. For this purpose, all other images were registered to the first *b* = 0 s/mm^2^ image. Images were then processed in an in-house developed Matlab script (R2020b, Mathworks, Natick, MA, USA) for diffusion-tensor fitting. Next, the slice in which the maximum visible intramuscular length of the SSP tendon was visible was located in the *b* = 0 s/mm^2^ images. PAs were derived by projecting the first diffusion tensor Eigenvector onto the transverse imaging plane and computing the angle relative to the SSP tendon, which was manually drawn on the *b* = 0 s/mm^2^ image by a fellowship-trained radiologist (A.A.M.) (Fig. 2). Images were then analyzed by three fellowship-trained radiologists (A.A.M., G.W.K., and S.S.G.) who were blinded to all participant data and read images in random order on a dedicated workstation. Two freehand regions of interest (ROIs) were drawn by each reader on the relevant slice of maximum tendon visualization by the readers in the computed PA maps using ITK-SNAP (v4.0.2) [23]. The ROIs encircled the anterior and posterior SSP muscle bundle respectively (hereafter, ROI_ant_ and ROI_post_), while surrounding structures and the SSP tendon itself were carefully excluded. PA measurements of one reader (A.A.M.) were repeated after a three-week interval to assess intra-reader agreement.

### Tractography

DTI tractography was performed solely for visualization purposes and was not a primary endpoint of this study. The motion-corrected STEAM dataset of an exemplary subject at a single time point was processed in MRtrix3 [24]. After estimating a mask, a deterministic tensor algorithm was applied for tractography. At each streamline step, the diffusion tensor was fitted to the local (trilinear-interpolated) diffusion data, and the trajectory was determined by the principal eigenvector of that tensor. Seed points were set randomly, and a total of 100.000 streamlines were generated, whereby a minimum length of 50 mm was enforced, and the angular cutoff was set to 20°.

### Statistical analysis

SPSS (v29, IBM Corp., Armonk, USA) was used for all statistical analyses. The Shapiro–Wilk test was used to test for the normal distribution of continuous data. Data are presented as mean ± standard deviation if normally distributed and as median with range in parentheses in cases of non-normal distribution. PAs were averaged across examination time points and readers for further analysis. To assess the test-retest reliability, inter-reader agreement, and intra-reader agreement of PAs measured with DTI, intraclass correlation coefficients (ICCs) for absolute agreement were calculated using a two-way mixed model. Results were categorized according to Koo and Li [25]: ICC > 0.90: excellent; 0.75–0.90 = good; 0.50–0.75 = moderate; < 0.50 = poor. *p*-values below an alpha level of 0.05 were considered significant.

## Results

### Participants

The mean age of the ten subjects was 32.0 ± 4.7 years (five females; body mass index 23.0 ± 2.6 kg/m^2^). Eight out of ten volunteers reported participating in recreational sports activities, while four participants mentioned their past involvement in competitive sports (soccer: *n* = 3, volleyball: *n* = 1). Qualitative image analysis of the anatomical MRI sequences did not reveal incidental findings or pathologies.

### Quantitative image analysis

**Fig. 3:**
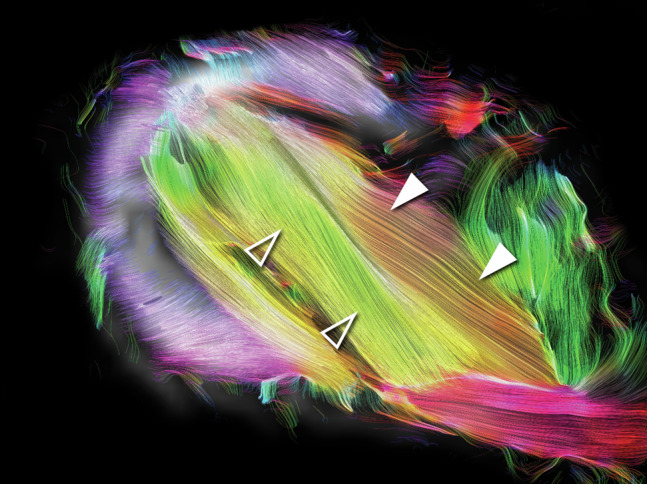
Axial STEAM DTI slice with color-coded tractography streamlines of the right shoulder in a 37-year-old male volunteer. The bipennate anterior portion of the SSP muscle (solid arrowheads) features a different configuration of fiber bundles adjacent to the tendon (solid and outline arrowheads)

The mean PA in the anterior SSP was 15.6 ± 2.1°, PA while for the posteriorly drawn ROI (ROI_post_), the PA was 10.7 ± 0.9°. Individual measurements obtained in MRI for each reader and examination session can be found in Table 2. PAs showed good to excellent test-retest reliability (ICC: 0.856–0.945). Inter-reader agreement was good to excellent (ICC: 0.796–0.955). Similarly, ICCs for intra-reader agreements ranged from good to excellent (ICC: 0.804–0.955, Table 3). An axial cut with color-coded tractography streamlines depicts the different configurations of the fiber bundles in the anterior SSP muscle bundle (Fig. 3).

**Table 2:** PA measurements for the two ROIs manually drawn anteriorly (ROI_ant_) and posteriorly (ROI_post_) in the PA maps derived from MRI

	Reader 1	Reader 2	Reader 3	
PA ROI_ant_ (°)				
				Inter-reader agreement^a^
Examination session 1	14.3 ± 1.9	15.6 ± 2.4	15.4 ± 2.4	0.865 (0.621–0.963)
Examination session 2	15.1 ± 2.3	16.0 ± 1.5	15.8 ± 1.2	0.955 (0.852–0.988)
Examination session 3	15.3 ± 1.6	16.6 ± 1.7	16.5 ± 1.5	0.878 (0.608–0.968)
Test-retest reliability^a^	0.899 (0.716–0.972)	0.914 (0.751–0.977)	0.888 (0.680–0.969)	
PA ROI_post_ (°)				
				Inter-reader agreement^a^
Examination session 1	10.4 ± 0.9	11.2 ± 1.5	10.2 ± 1.3	0.796 (0.424–0.944)
Examination session 2	10.6 ± 1.0	10.8 ± 1.0	10.3 ± 1.9	0.905 (0.727–0.974)
Examination session 3	10.8 ± 1.8	11.0 ± 1.2	10.6 ± 0.7	0.863 (0.613–0.962)
Test-retest reliability^a^	0.945 (0.825–0.985)	0.856 (0.583–0.961)	0.798 (0.422–0.945)	

Measurements are given for the three readers and each MRI examination session, respectively. Test-retest reliability and inter-reader agreement were assessed using ICCs

^a^ Test-retest reliability and inter-reader agreement given by ICCs with 95% confidence intervals (CIs) in parentheses

**Table 3:** PA measurements for the two ROIs manually drawn anteriorly (ROI_ant_) and posteriorly (ROI_post_) in the PA maps derived from MRI

	First reading session	Second reading session	
PA ROI_ant_ (°)			
			Intra-reader agreement^a^
Examination session 1	14.3 ± 1.9	14.3 ± 2.7	0.825 (0.253–0.957)
Examination session 2	15.1 ± 2.3	14.2 ± 2.3	0.922 (0.486–0.983)
Examination session 3	15.3 ± 1.6	16.8 ± 1.7	0.955 (0.808–0.989)
PA ROI_post_ (°)			
Examination session 1	10.4 ± 0.9	10.1 ± 0.7	0.916 (0.443–0.982)
Examination session 2	10.6 ± 1.0	10.7 ± 2.1	0.891 (0.553–0.973)
Examination session 3	10.8 ± 1.8	11.0 ± 1.5	0.804 (0.181–0.952)

Measurements are given for the two reading sessions of one reader. Intra-reader agreement was assessed using ICCs

^a^ Intra-reader agreement given by ICCs with 95% CIs in parentheses

## Discussion

In this study, a method to derive PAs of the SSP muscle from DTI was developed and validated. The proposed approach demonstrated good to excellent test-retest reliability, inter-reader agreement, and intra-reader agreement.

The first main finding was that PAs of the SSP as measured with the DTI methods were within the range of those described in the literature [8–11], which depend on the exact anatomical localization within the muscle [9]. The present study measured PAs of the anterior portion and posterior portion (~ 16° and ~ 11°, respectively) that were slightly higher for the anterior SSP than in a cadaver study by Kim et al, reporting mean values of ~ 12° for the middle part of the anterior and posterior SSP muscle, respectively [9]. This might be explained by the higher age of the examined specimens in their study compared to our in vivo population (mean age of 62 years vs 32 years, respectively), since PA is known to physiologically decrease with increasing age [26].

This study calculated PAs of the anterior SSP muscle bundle in a 2D manner from the principal Eigenvector in relation to the intramuscular tendon, which was also described by Bolsterlee et al [15] and Takahashi et al [27]. In contrast to our study, these authors used a DTI tractography framework that reflects myocyte orientation in 3D space [15, 18, 27]. We acknowledge that 2D muscle architecture analyses only consider a fraction of the fascicles that intersect the imaging plane. Therefore, these analyses exhibit limited comparability to the measurements obtained from 3D analyses, as fascicles form highly variable and complex structures within the muscle. However, the methodology used in our study offers a significant advantage over those found in the current literature due to its high degree of standardization regarding the choice of imaging plane and overall lower complexity, which is corroborated by the good to excellent test-retest reliability, inter-reader agreement, and intra-reader agreement of the method. This might make it a promising alternative for practical implementation, since DTI muscle tractography and PA measurements have been investigated for at least two decades but have not yet moved out of the research domain into a clinical routine [12].

Moreover, ICCs of MRI-derived PA measurements surpassed those reported in a study by Kim et al, in which the authors used ultrasound to measure PAs of the SSP muscle [9]. This may be explained by the operator’s dependence on ultrasound measurements: due to differences in probe angulation and pressure application during the examination, PA measurements are reported to be susceptible to errors, particularly in the rotator cuff muscles [7, 28].

This study is not without limitations. First, it should be noted that the present study was conducted on healthy volunteers, which precludes any inferences regarding the translation of our results to patients with rotator cuff tears. Second, the study is limited by a small sample size which may affect the interpretability of our results, even though the MRI scans were repeated three times to assess test-retest reliability.

## Conclusion

We presented a novel low-complexity framework for measuring PAs of the SSP muscle using DTI with a highly standardized approach. A good to excellent test-retest reliability, inter-reader agreement, and intra-reader agreement were observed. As quantitative biomarkers are increasingly used to assess rotator cuff pathology, our approach may have the potential for clinical translation to complement a comprehensive assessment of quantitative shoulder MRI.

## Data Availability

The data underlying this article cannot be shared publicly due to regulatory constraints. The data will be shared on reasonable request to the corresponding author.

## Abbreviations

CI: Confidence interval
DTI: Diffusion tensor imaging
ICC: Intraclass correlation coefficient
PA: Pennation angle
SSP: Supraspinatus
